# Experience with the Jungfrau-1M detector at Diamond Light Source

**DOI:** 10.1107/S1600577526000342

**Published:** 2026-02-17

**Authors:** John Matheson, Danny Axford, Anna Bergamaschi, Maria Carulla, Nicholas Devenish, Noemi Frisina, Viktoria Hinger, Vadym Kedych, Christopher Lane, Aldo Mozzanica, Eva Gimenez-Navarro, James O’Hea, Dominic Oram, Robin L. Owen, David Perl, Adam Prescott, Bernd Schmitt, Shane Scully, Adam Taylor, Gary Yendell, Graeme Winter

**Affiliations:** a Diamond Light Source Harwell Science and Innovation Campus OxfordshireOX11 0DE United Kingdom; b Paul Scherrer Institut Forschungsstrasse 111 5232Villigen PSI Switzerland; c Argonne National Laboratory 9700 S. Cass Ave., Building 436E Lemont IL60439 USA; University of Essex, United Kingdom

**Keywords:** integrating detectors, macromolecular crystallography, synchrotron science

## Abstract

A Jungfrau-1M detector has undergone testing at Diamond Light Source. The Jungfrau series of detectors from PSI use integration and adaptive gain, to offer very high frame rate and dynamic range, suitable for high-flux and time-resolved measurements. They are becoming more widely used, to take advantage of increasing light source brightness. We report on our experiences in testing the performance of a Jungfrau-1M without illumination, with a laboratory X-ray tube and on a microfocus beamline. The Jungfrau-1M was found to be able to resolve single photons in the laboratory and on the beamline. It was confirmed that range switching from high to intermediate gain is associated with a discontinuity in the detector response. Two methods of dark frame subtraction were compared for their effect on minimizing this discontinuity. The Jungfrau-1M was found to be very effective for recording macromolecular crystallography diffraction patterns, with no apparent detriment from the discontinuity. The Diamond machine will be upgraded in 2028–9 and will operate at significantly higher flux than at present, necessitating increased use of integrating detectors, such as Jungfrau, in the future.

## Abstract 2

A Jungfrau-1M integrating detector was characterized at Diamond Light Source and found to be effective for recording macromolecular crystallography diffraction patterns. Parameters for operation and data collection were explored and characterized. The Diamond facility will be upgraded and integrating detectors, such as Jungfrau, will be used more widely in the future.

## 1. Introduction

**Figure 1:**
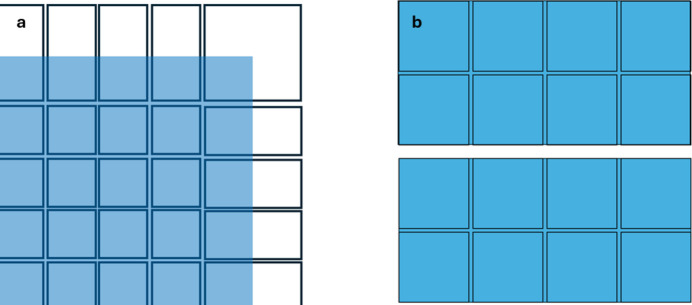
(*a*) Schematic of the mapping of the ASIC (blue) to the sensor, showing the sensor ‘big pixels’ at the periphery of the ASIC. (*b*) Tiling of ASICs in a 1M detector. See text for details.

The Jungfrau-1M (*i.e.* 1 Mpixel) detector has been developed by the Paul Scherrer Institute (PSI), Detectors Group. It is part of a family of detectors, based on tiling arrays of 0.5 Mpixel modules (Mozzanica *et al.*, 2016 ▸). Each module consists of a 4 by 2 array of readout integrated circuits (ASICs), bump-bonded to a single pixelated silicon sensor. ASIC pixels are 75 µm square. Sensor pixels are also 75 µm square, except around the inner edges of the ASICs, where the sensor pixels are larger to allow tiling space between the ASICs, without an insensitive region. Inner edge pixels are doubled in length and inner corner pixels are quad sized, as shown in Fig. 1 ▸(*a*). A single module contains 4 ASICs by 2 ASICs, *i.e.* 1024 by 512 pixels, as shown in Fig. 1 ▸(*b*). The gaps between ASICs are equivalent to two 75 µm pixels. The vertical gap between adjacent modules is equivalent to 36 pixels (at 75 µm), to allow for wire bonding along the ASIC outer edges. Where multiple 1M units are tiled together into a larger array, the horizontal gap between modules is 8 pixels.

**Figure 2:**
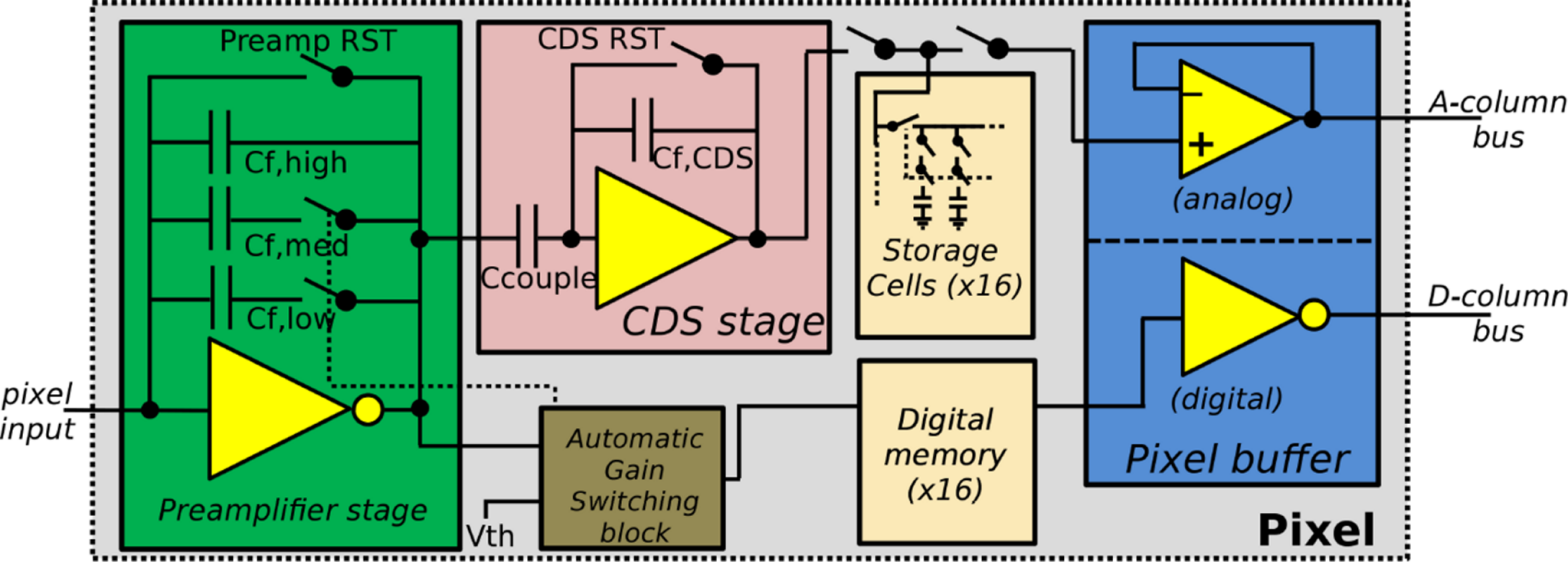
Schematic block diagram of a Jungfrau ASIC pixel.

Jungfrau has been designed to be capable of recording a higher photon flux than that possible using photon counting. This is achieved by integrating the charge from the sensor over a given time period, and by the use of dynamic gain switching. Gain switching allows sequential selection of three feedback capacitors within the charge-integrating preamplifier of each pixel, according to the magnitude of charge from the sensor. Fig. 2 ▸ shows a schematic layout of a Jungfrau channel readout chain (Smith *et al.*, 2015 ▸). Bigger feedback capacitors imply more noise, but the switching occurs at points that ensure the electronic noise is always less than the Poisson noise of the incident photons. The imaging performance is not expected to be significantly poorer than that from a counting detector (Redford *et al.*, 2016 ▸). This work used a detector based on Jungfrau V1.0 ASICs, which will eventually be superseded by the Jungfrau V1.2. The new version ASIC is currently under test, but the ASICs have not yet been assembled into large-area arrays.

Gain settings are denoted by G0 (high gain), G1 (medium gain) and G2 (low gain). Jungfrau is supplied with calibration maps, typical calibrations being: G0 = 41.1 ADC units (ADCU) per keV, G1 = −1.36 ADCU per keV and G2 = −0.107 ADCU per keV. The negative gain in the higher ranges is a result of the details of the chip architecture and is accounted for during image correction (Redford *et al.*, 2016 ▸). The calibration is carried out using fluorescence X-rays in G0, a pulsed voltage applied to the sensor backplane in G1 and an on-chip current source in G2 (Redford *et al.*, 2018 ▸). The detector switches from G0 to G1 near 25 photons, from G1 to G2 near 750 photons and the limit in G2 is near 1.0 × 10^4^ photons, per pixel per frame, at 12.4 keV. The range maxima and gain switching points depend on photon energy.

Charge integration implies that, in the absence of illumination, the detector integrates any leakage currents from the sensor and the front-end device of the ASIC. Dark runs must be recorded under the same conditions as the experimental data capture and the dark pixel DC values (termed ‘pedestals’) subtracted from the pixel values under illumination. Dark runs must be carried out for each of the three gain ranges; however, the detector will not switch gain in the absence of illumination and must be manually switched into medium or low gain for pedestal runs. This mode of operation is termed ‘forced gain mode’ and is discussed in more detail later.

The Jungfrau was originally designed for use at SwissFEL, where the short beam pulses allow for correspondingly short integration times. At a synchrotron light source, longer integration times are typically used, and the detector leakage current must be suppressed by cooling (Redford *et al.*, 2018 ▸). The longer the integration time used, the more important are cooling and temperature stability. On synchrotron light sources, 1 ms integration/1000 fps (frames per second), or 0.5 ms integration/2000 fps, would be typical. Shorter integration periods might be used for techniques such as beam chopping (Osawa *et al.*, 2017 ▸). PSI recommends operating the Jungfrau with water and antifreeze mixture near −12°C, with temperature stability near ±0.1°C. To avoid condensation, the Jungfrau-1M was operated inside an enclosure, flushed with dry air.

The analogue output of each pixel is digitized by a 14-bit ADC and two additional bits encode the gain setting for each pixel. The maximum frame rate is 2200 fps, leading to a data rate of 18 Gb s^−1^ per module. Each module uses two 10 Gbit s^−1^ multimode optical links. In this work, the four 10 Gb s^−1^ outputs were combined into a single 40 Gbit s^−1^ data stream, via a network switch (Mellanox SN2100). The 40 Gbit s^−1^ data were input to a server running the data acquisition software, which was based on the *SLS Detector Package* from PSI. The data rate from the 1M unit was near the limit of the network bandwidth from the beamline to the data centre. The use of integrating detectors in general will be a significant challenge for networking in the future, with increasing use of data correction and analysis, in parallel to data collection, to reduce the bandwidth requirements (Leonarski *et al.*, 2020 ▸; Leonarski *et al.*, 2023 ▸).

Jungfrau detectors have been made with varying numbers of modules, up to a maximum of 16M, and have been deployed on XFEL and synchrotron beamlines. The high frame rate makes Jungfrau a good choice for time-resolved macromolecular crystallography and serial crystallography (Tolstikova *et al.*, 2019 ▸). Within Diamond Light Source (DLS), the Jungfrau-1M was evaluated in the Laboratory of the Diamond Detectors Group and on the Microfocus Macromolecular Crystallography beamline, I24. This work was intended as a precursor to the delivery of a 9M version, delivered in summer 2025 and currently being commissioned on I24.

## 2. Dark image measurements

### 2.1. Pedestals and noise dependence on temperature

**Figure 3:**
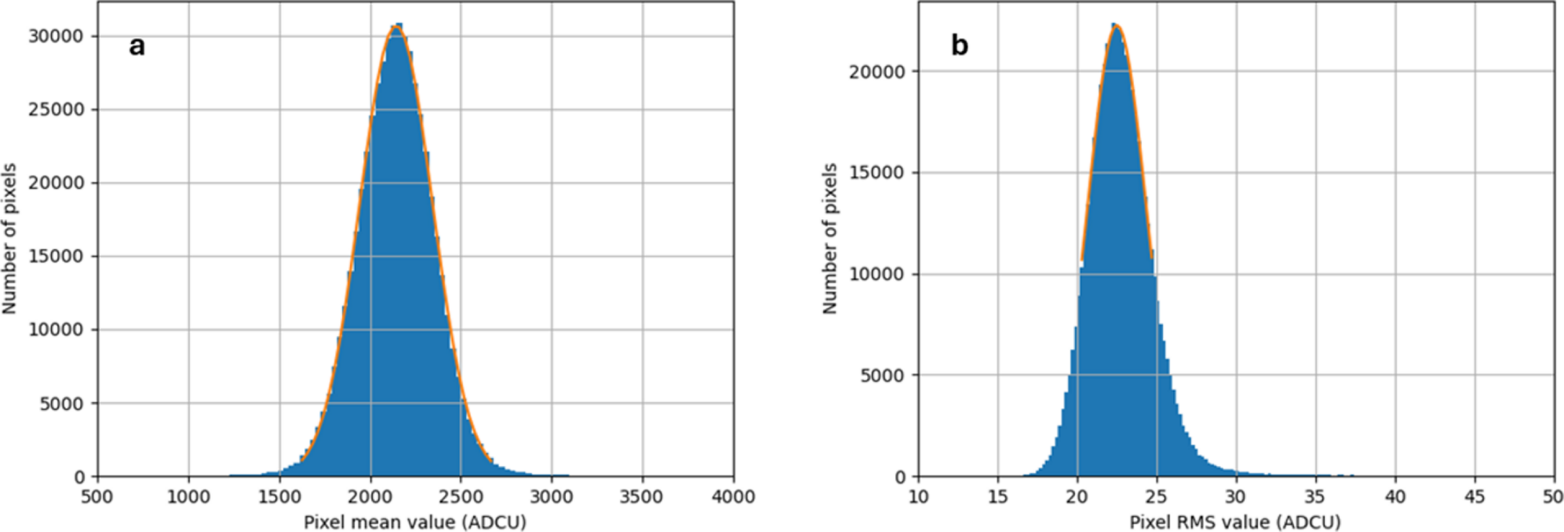
(*a*) Distribution of pixel mean value (*i.e.* pedestal) at 1 ms integration time in G0. (*b*) Distribution of pixel RMS deviation at 1 ms integration time in G0. Coolant temperature was −11°C. (G0 gain is near 41.1 ADCU per keV.)

Dark images from the Jungfrau-1M (in G0) were recorded and the pixel values were histogrammed. The most probable pedestal value was found by a Gaussian fit to the resulting distribution. An example fit is shown in Fig. 3 ▸(*a*). The standard deviation of each pixel over a stack of 100 images was calculated, to give a measure of its noise. The standard deviations of all the pixels were histogrammed and the most probable value found using a Gaussian fit. An example fit is shown in Fig. 3 ▸(*b*). The noise distribution over the pixels was skewed, with a tail of higher noise pixels. The fit was made to the upper half of the distribution, which is a reasonable estimate of the most probable value, but is equivalent to cutting those pixels with the highest noise values.

**Figure 4:**
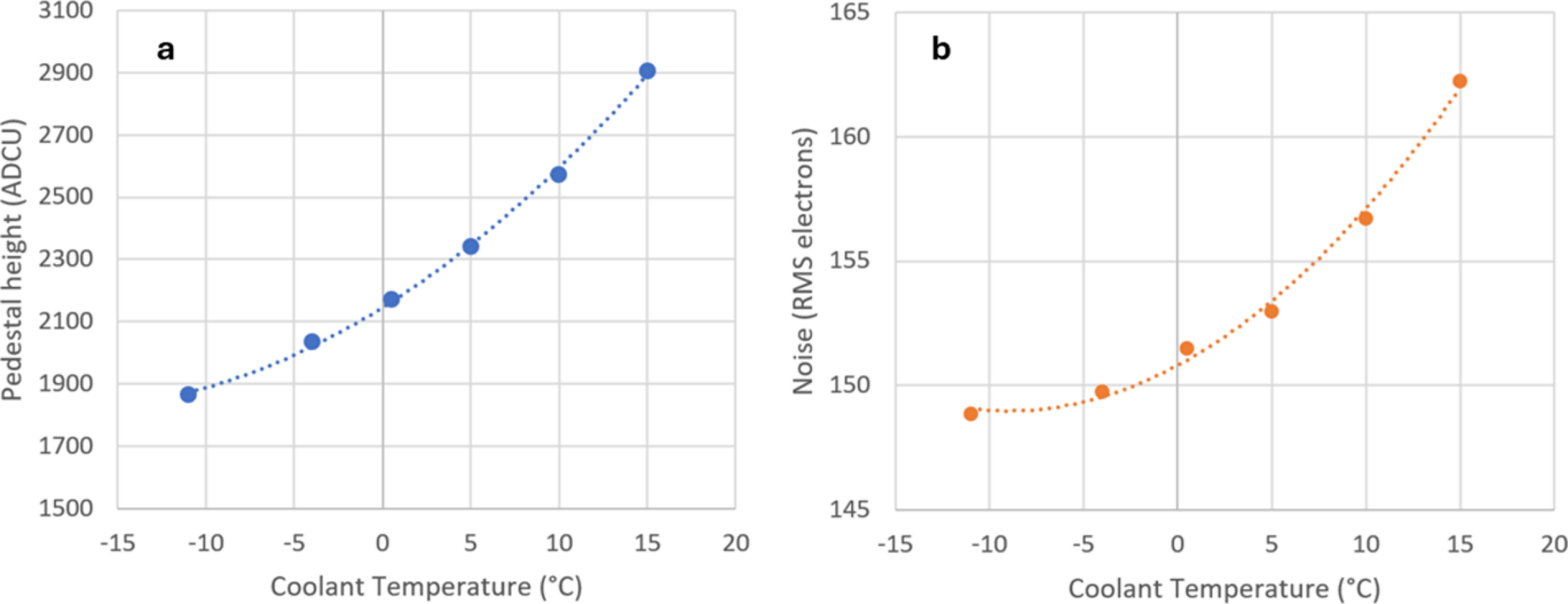
(*a*) Pedestal height versus coolant temperature at 1 ms integration time in G0. The maximum ADC value is near 16 K. (*b*) Dark noise (mean of all pixel standard deviations) versus coolant temperature at 1 ms integration time in G0. (Assuming G0 gain of 41.1 ADCU per keV and 3.62 eV per electron hole pair in silicon.)

The most probable pedestal and noise values were determined as a function of integration time, temperature and frame rate. The effect of coolant temperature on pedestal height is shown in Fig. 4 ▸(*a*), for a representative integration time of 1 ms. [The fit lines in Figs. 4 ▸(*a*) and 4 ▸(*b*) are quadratic and are intended only to aid interpretation of the data.] The cooling was found to reduce the pedestal height in G0, which results in a more effective use of the dynamic range of the front-end. If a significant fraction of the preamplifier range in G0 were to be taken up by the dark pedestals, the dynamic range with single-photon resolution remaining for signal would be reduced. The 1050 ADCU improvement in pedestal height found by lowering the temperature from 15°C to −12°C was modest, compared with the available dynamic range near 16 K ADCU. Most of this benefit was obtained for a coolant temperature of −5°C, so that cooling to −10°C was deemed more than adequate for operating the detector.

The electronic noise (*i.e.* the random variation in the pedestal value) was also found to be reduced by decreasing the coolant temperature, as shown in Fig. 4 ▸(*b*). The rate of change of the noise with decreasing temperature was less significant, below 0°C. Overall, the detector works well, operated with the coolant close to −10°C, with only a little additional benefit in cooling to lower temperatures, so that the standard recommendation of −12°C from PSI may be used with confidence. The temperature of the module readout board (ROB) was near +12°C under these operating conditions, based on the ROB internal sensors. No monitor for the sensor temperature is incorporated in the Jungfrau-1M design, with the sensor temperature expected to be close to the coolant temperature (to 1–2°C). In this work, a −10°C coolant setting was used throughout, unless explicitly stated otherwise. After the detector had been in use for some time, radiation damage was observable in some areas as slightly increased pedestal and noise values, increasing the skewness of the pedestal and noise distributions. These changes are minimized by operation at low temperature and short integration times. A few tens of pixels were seen with a noise which was large enough to mimic a beamline photon (12.4 keV at beamline I24) and these were masked out.

### 2.2. Noise as a function of frame rate and integration time

**Figure 5:**
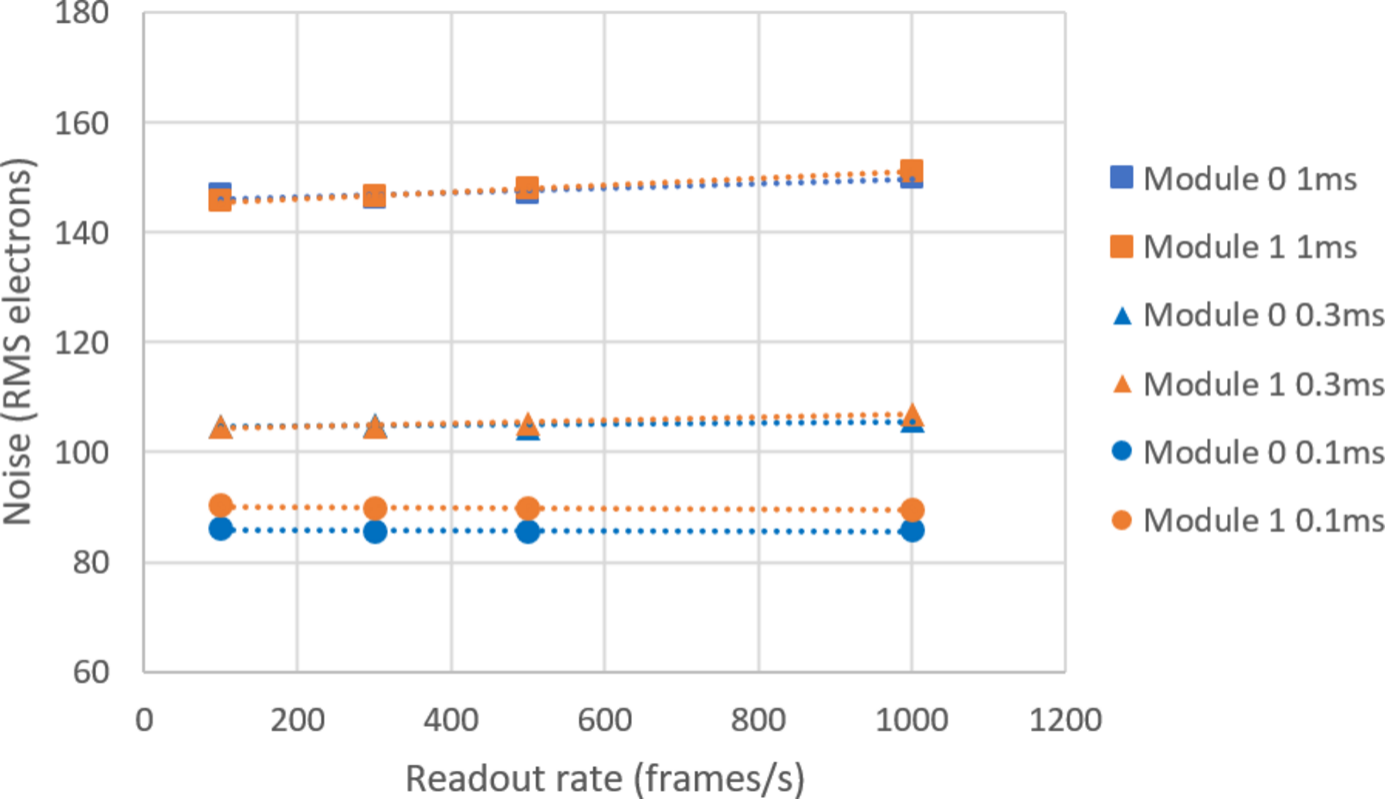
Electronic noise as a function of frame rate, for three different integration times. (Assuming G0 gain of 41.1 ADCU per keV and 3.62 eV per electron hole pair in silicon.)

The electronic noise from the detector could potentially show some dependence on the frame rate. If this was significant, it could affect the settings that would maximize the detector signal-to-noise ratio. Dark frames were accumulated with a single integration time, varying the frame rate. This was repeated for three different integration times. Pixel noise was calculated as before. The results are presented in Fig. 5 ▸, which shows that the noise increased with integration time, as expected. The noise dependence on the frame rate was found to be a small effect.

On a synchrotron beamline (essentially a DC source), one would expect to use a short integration time to minimize the noise, which then requires a high frame rate to maximize the use of the available photons. A significant increase of noise with increasing frame rate might imply that there is some optimal trade-off between integration time and frame rate. At an integration time of 1 ms, there is some increase of noise with frame rate, but this is rather small. To obtain the best performance, it should suffice to run the detector at its maximum frame rate and to select the integration time accordingly, *i.e.* near 2000 fps and 0.5 ms, respectively.

### 2.3. Stability of dark measurements

**Figure 6:**
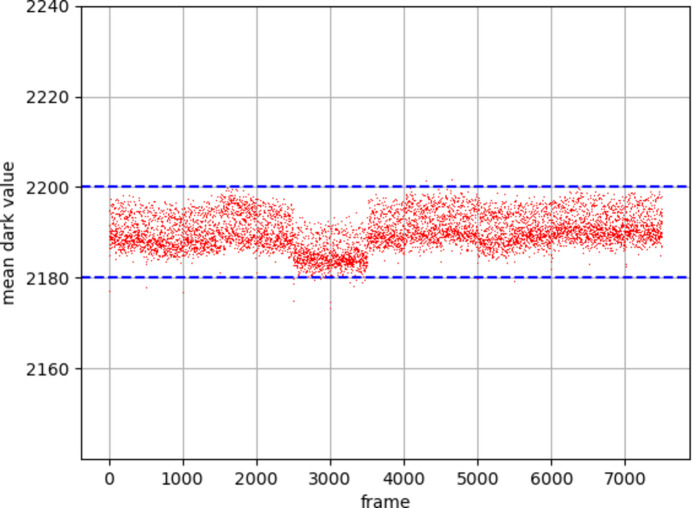
G0 mean pedestal value versus frame number, for a series of pedestal runs concatenated over 24 h. Integration time 0.5 ms, frame rate 2000 fps (G0 gain is 41.1 ADCU per keV).

The stability of the pedestal values with time is important for an integrating detector, as fluctuations could disrupt measurement of diffraction data. Multiple individual pedestal runs were recorded, interspersed between data taking runs on DLS beamline I24. Pedestal stability results for G1 and G2 are shown in Section 4.1. For G0, the mean pedestal value of each frame is shown versus time for a single ASIC in Fig. 6 ▸. The pedestal runs were taken irregularly over a period near 24 h and are shown concatenated; 500 samples are shown for each separate run. The detector was cooled and powered on for the entire period, but data were taken only during working hours. There are some small changes from run to run, which are visible in Fig. 6 ▸. These changes were present in all the ASICs of the detector and were likely a result of ambient temperature changes over the whole period. For example, frame 2500 was the start of the first pedestal run of the day, recorded immediately after an overnight break, over which no data had been taken. Frames 2500 to 3500 correspond to two pedestal runs immediately after the break. Although a jump can be seen, it is small compared with the 12.4 keV photon energy typically used for macromolecular crystallography (MX) measurements.

The pedestal values in G0 were found to be reproducible. The stability on the beamline was better than ±10 ADCU (as indicated by the dotted blue lines), equivalent to ±0.25 keV, over several hours. This suggests that the frequency of pedestal measurements does not need to be so high as to be a significant overhead on beam time, assuming that the ionizing dose to the detector is small over the same time frame.

## 3. Laboratory X-ray measurements with the Jungfrau-1M

### 3.1. Fluorescence X-ray measurements with the Jungfrau-1M

#### 3.1.1. Single-photon detection

**Figure 7:**
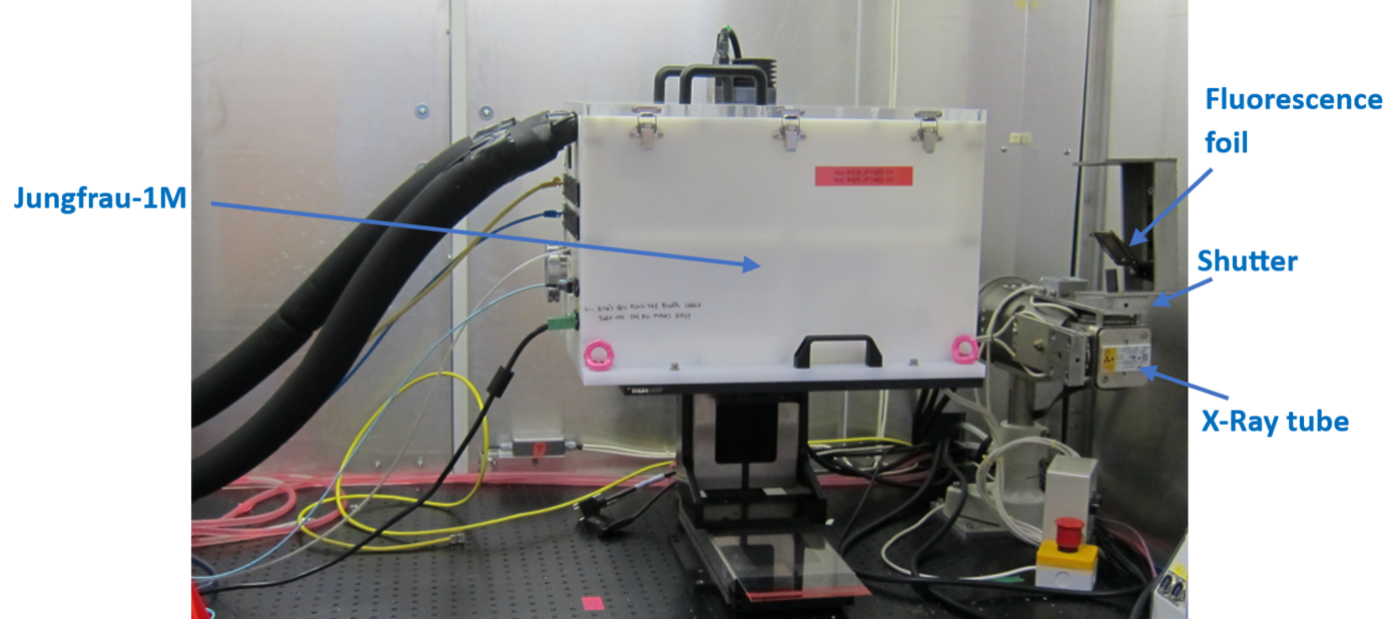
Arrangement for single-photon measurements.

To evaluate the detector for single-photon detection, it was set up, with a copper fluorescence foil, in the X-ray cabinet of the Diamond Detectors Group (this uses an X-ray tube with an Mo target). The detector was mounted on X–Y motion stages, at a distance near 20 cm from the foil. This arrangement is shown in Fig. 7 ▸. The use of fluorescence gave a defined photon energy, with reasonable uniformity at the detector and the tube current was set to give a sparse illumination of single photons, near 10^4^ photons mm^−2^ s^−1^.

In order to collect sparse hits and also to suppress pixel noise, a relatively short integration time of 100 µs was used initially. To correct the raw images from the detector, pedestal data were first subtracted and then each pixel corrected for its gain. Gain calibrations were those provided by PSI. The gain setting of each pixel in the raw image data was used to select the corresponding pedestals and calibrations, although all pixels remained in G0 in this case.

The effect of masking pixels with noise higher than a certain level was evaluated. The image, corrected for pedestals and gains, was multiplied by a mask containing ones for accepted pixels and zeros for rejected pixels. Acceptance criteria were applied to the standard deviation *s* of each pixel (*i.e.* its noise) over a stack of images. The mean μ and standard deviation σ of *s* were calculated over all pixels. Anomalously low (*i.e.* close to zero) noise most likely means a disconnected bump bond. Pixels with high noise may be mis-identified as signal. Lower and upper cuts were therefore placed on *s*. Because of the shape of the noise distribution, the upper cutoff between a good and a bad pixel was found to be very subjective. As an example, for 0.5 ms integration time, an absolute cut on the noise, equivalent to 0.1 beamline photon (*i.e.* 1.2 keV or 49 ADCU) led to 0.1% of pixels being rejected. Mis-identification of pixel noise as signal is less probable than with a counting detector due to the averaging effect of the integration, and there was no significant effect on the spectrum mean or width from applying the upper cut.

The corrected image, representing for each pixel the actual energy deposited in keV, was calculated according to: Corrected image = Pixel mask × [(Raw image − Pedestal image) / Calibration (ADCU per keV)].

**Figure 8:**
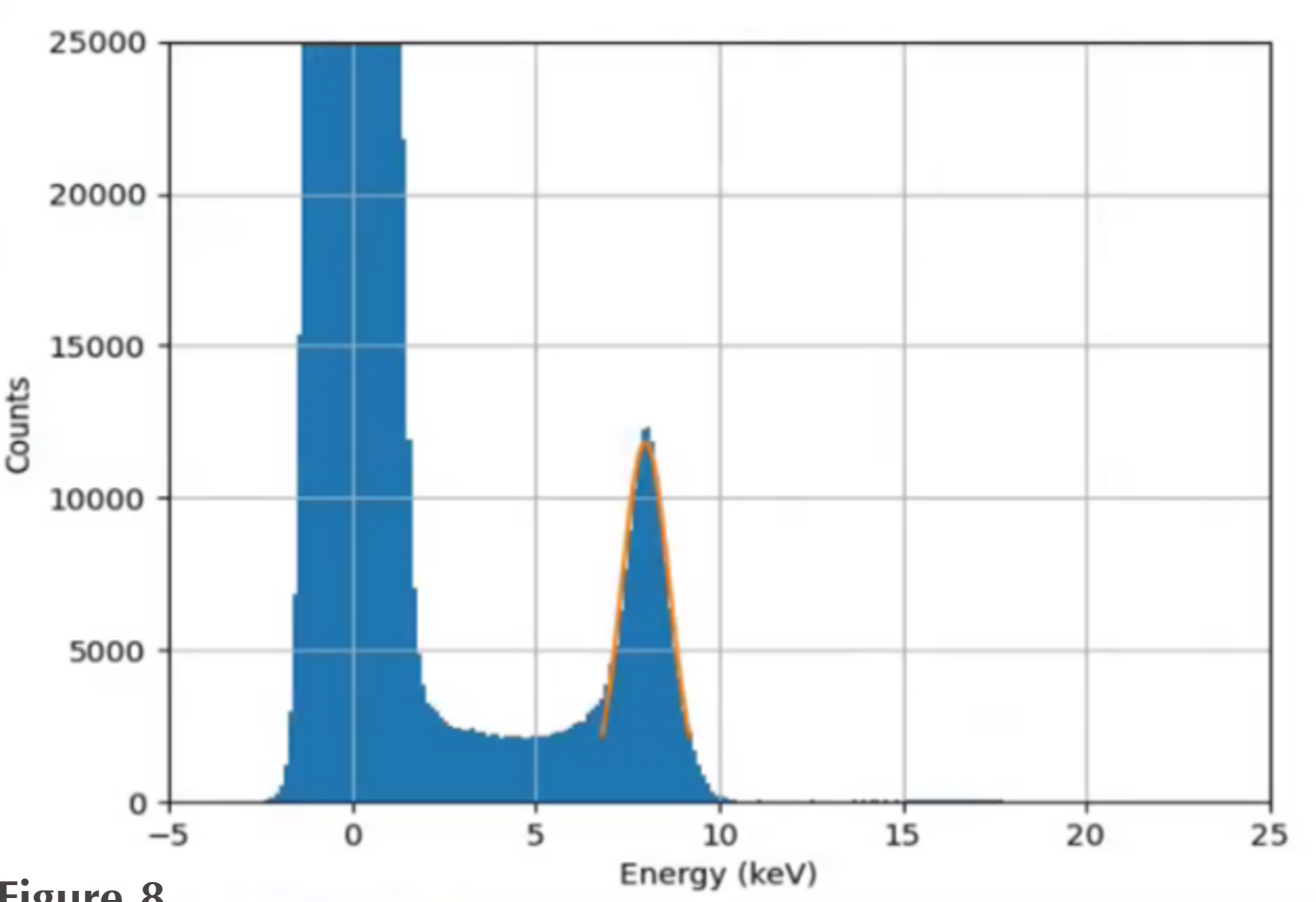
Histogram of corrected data over all pixels in one module. A Gaussian was used to fit the photopeak, as shown in orange (see text).

Single-photon hits after correction and calibration are shown histogrammed in Fig. 8 ▸.

The photopeak in the spectrum was fitted with a single Gaussian, to extract the mean and standard deviation. The standard deviation was 645 ± 5 eV, which was more than that from the electronic noise alone, due to effects such as charge sharing and partial charge collection. The mean value of the fit was 7.996 ± 0.003 keV, close to the expected value for the copper *K*α line at 8.04 keV. The error on this value was obtained by dividing up the data into three stacks of frames and performing the fit three times, taking the standard deviation of the three mean values to be the random error on the measurement. The *K*β line at 8.91 keV was not readily discernible from the histogram, in contrast to the findings of Redford *et al.* (2016 ▸), in which a shorter integration time was used. The extracted mean value above was slightly lower than the expected weighted mean of the *K*α and *K*β lines, likely due to charge sharing effects. However the results do demonstrate a clear single-photon detection capability in G0, which is important for the application to MX.

#### 3.1.2. Single-photon detection with different fluorescence foils

**Figure 9:**
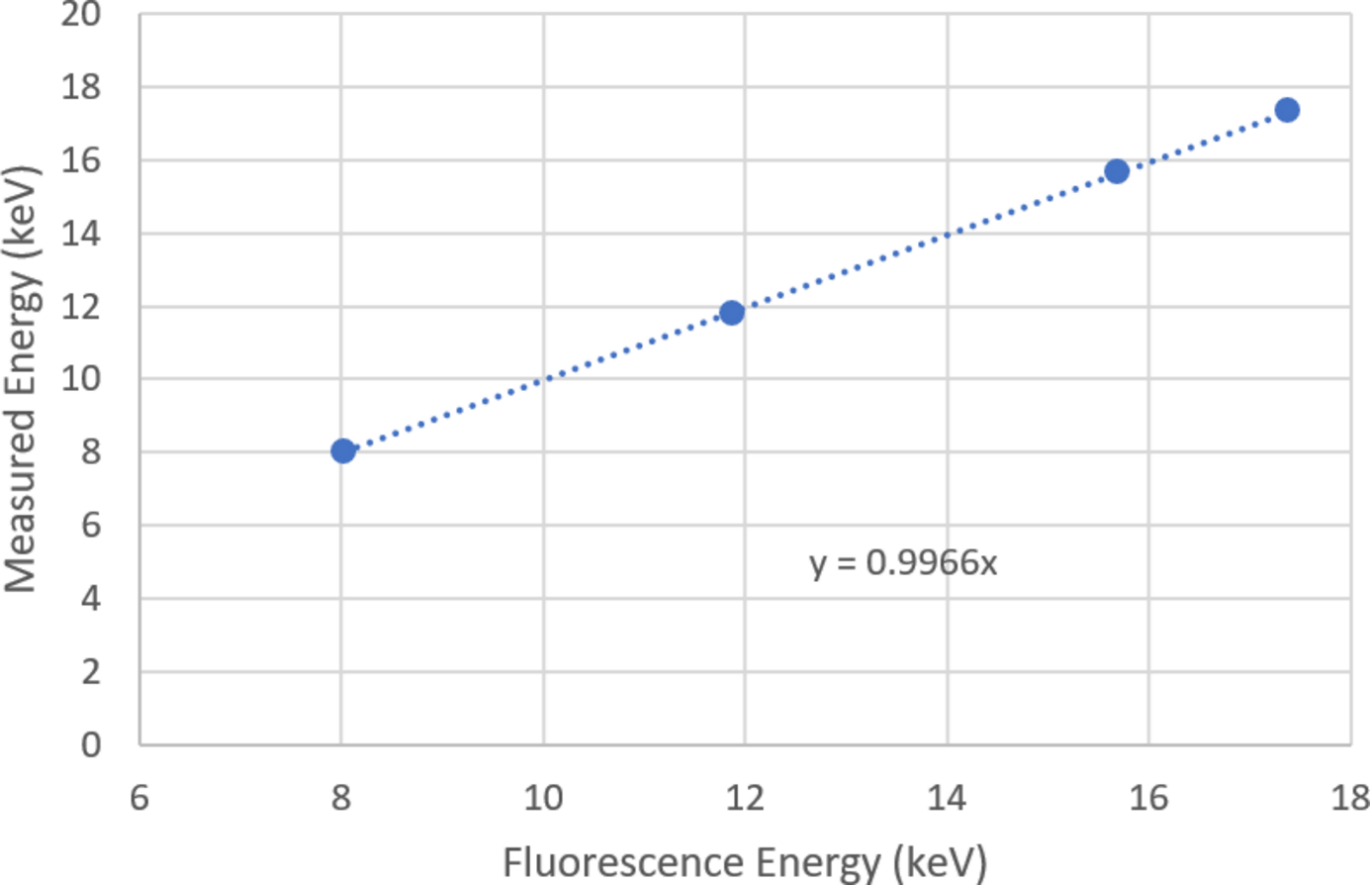
Calibration check – photon energy measurement from the Jungfrau, using four fluorescence foils to give a range of incident photon energies.

Data were recorded with a copper fluorescence foil, as detailed above. Measurements were also made with foils fluorescing at higher energy (KBr, Zr and Mo). The peak position was reconstructed as 17.324 ± 0.07 keV for Mo, compared with 17.374 keV (Mo *K*α) and 15.675 ± 0.017 keV for Zr, compared with 15.691 keV (Zr *K*α). The slightly lower measured energies may be due to charge sharing between pixels and the different models used in calibration and data fitting. A fit taking account of charge sharing is presented by Redford *et al.* (2018 ▸). Fig. 9 ▸ shows a calibration check using data from all the foils used.

The G0 calibration supplied by PSI was found to be accurate, with good linearity for sparse illumination with single fluorescence photons. Single-photon detection is important for MX measurements, in order to resolve the weakest reflections from a sample.

### 3.2. Direct beam measurements with the Jungfrau-1M

#### 3.2.1. Check of G1 calibration relative to G0 calibration

The gain switching of the Jungfrau-1M was investigated by using direct illumination from the laboratory X-ray tube, to give a higher flux than that from fluorescence foils. The distance between the detector and the tube was made as large as practically possible within the cabinet (near 90 cm separation), to make the illumination more uniform and to minimize radiation damage.

**Figure 10:**
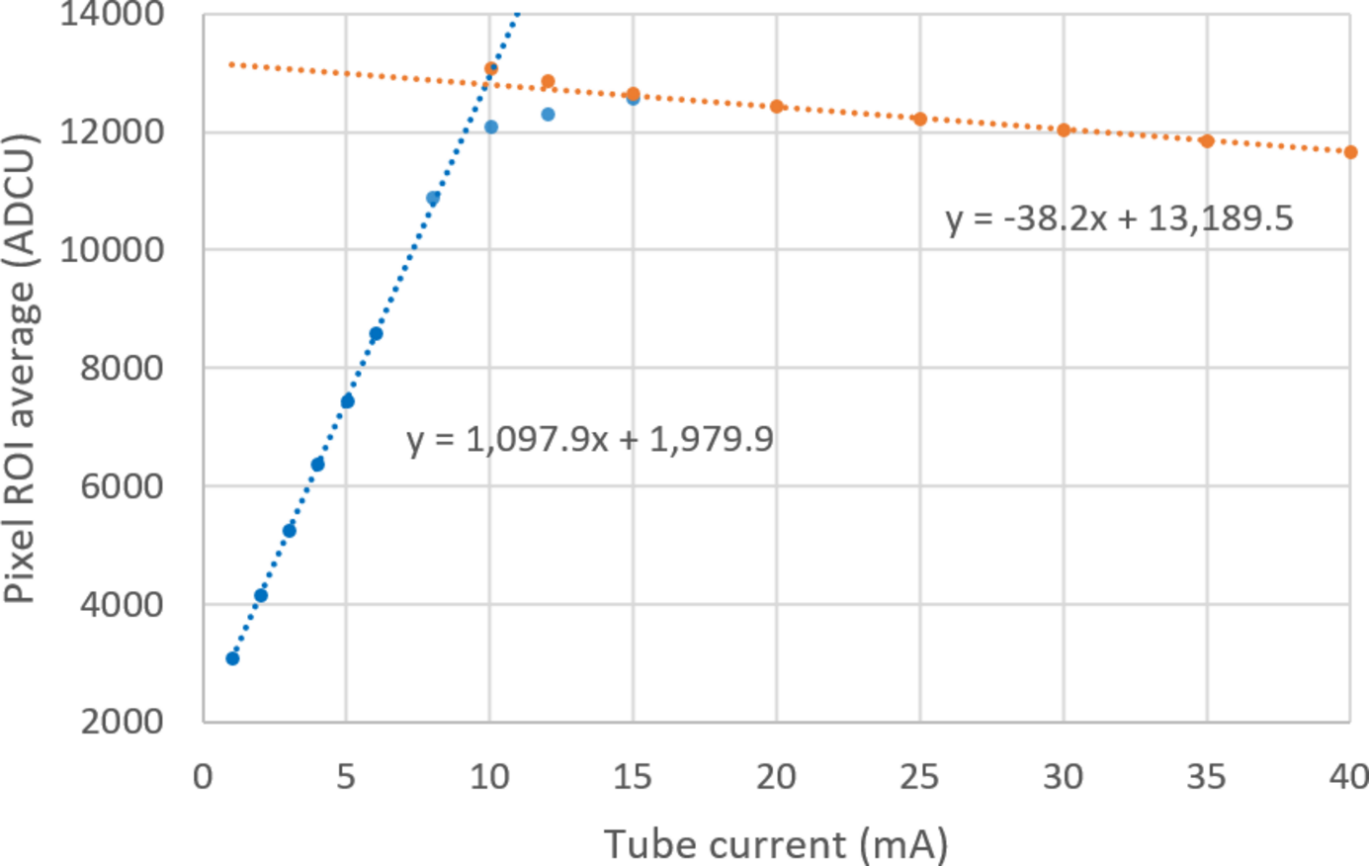
Output response in raw ADCU versus X-ray tube current. Note that the gain is positive for G0 (blue) and negative for G1 (orange), by design. Linear fits are shown to the two gain regions, excluding points from the switching region.

A relative measurement of the ratio of gains between G0 and G1 was made, using unfiltered beam rather than monoenergetic photons. The total charge deposited was nonetheless expected to be linear with the X-ray tube current. The analysis was carried out on an area of 200 by 200 pixels, near the centre of the X-ray spot. For each tube current, the pixel readings over this area were histogrammed, and the resulting distribution fitted with a Gaussian. The first step in the analysis was to determine which pixels had switched from G0 to G1. The switched pixels and unswitched pixels were analysed separately and their signal was plotted against the X-ray tube current (Fig. 10 ▸). In the switching region (8–15 mA), the variation of the number of incident photons, the variation of the energy deposited per photon and the selection introduced by the gain switching circuit, cause the G0 curve (which has a positive gain coefficient) to bend downwards and the G1 curve (which has a negative gain coefficient) to bend upwards. Because each point in the figure is an average over 200 × 200 pixels and 10 frames, the errors are smaller than the plot symbols.

Using the gains from PSI for this module, the mean gain over all pixels was 41.36 ADCU per keV for G0 and −1.36 ADCU per keV for G1. The errors on these values were ±1%, based on the work of Redford *et al.* (2018 ▸). This gives a ratio of −30.41 ± 0.43. In Fig. 10 ▸, the region where the gain switching occurred was excluded. The fit was found to have a small dependence on the choice of included data points and this was used to make an error estimate. The error on the fit was estimated to be ±3% for G0 and ±2% for G1, based on varying the number of points included. This results in an average gain ratio of −28.7 ± 1.1. The calibration in G1 measured at DLS is therefore consistent with the supplied value, within experimental errors.

#### 3.2.2. Linearity at the point of switching

To characterize the linearity of the Jungfrau-1M at the point of switching from G0 to G1, the direct beam from the X-ray tube was used. The detector box was aligned to centralize the beam spot on the upper module, flood illuminating it. There was marginally less than complete coverage of the sensors by the beam. The lower module was masked by a lead sheet. Only upper module data are shown.

In order to subtract the background from the images, dark runs were first recorded. G0 dark frames were recorded at 1000 fps. G1 and G2 dark frames were recorded in forced gain mode, which entails an increase in the power consumption of the ASIC. This may lead to an increase in the temperature and a small but measurable power rail voltage drop of the ASIC, and so introduce a statistical bias to the pedestals themselves. The technique used in forced gain pedestal measurements is therefore important. Two methods were used. In ‘continuous mode’, forced gain measurements were run with all frames in forced gain, but using a reduced frame rate of 100 fps, to minimize the heating and power rail effects. In ‘interspersed mode’, forced gain measurements were run with every 20th frame in forced gain, and the rest in G0, allowing the use of a frame rate of 1000 fps, to match the experimental conditions more closely, whilst still minimizing any temperature rise.

For each interspersed mode run, 4000 images were recorded, with every 20th image being a forced gain measurement (a total of 400 images in forced gain). The first 200 forced gain frames were taken at G1 and the last 200 were taken at G2. Pedestal values were calculated as the average of the 200 G1 frames and these were subtracted from those pixels in each illuminated frame which had switched into G1. Illuminated data were recorded at 1000 fps. The integration time was 1 ms throughout.

**Figure 11:**
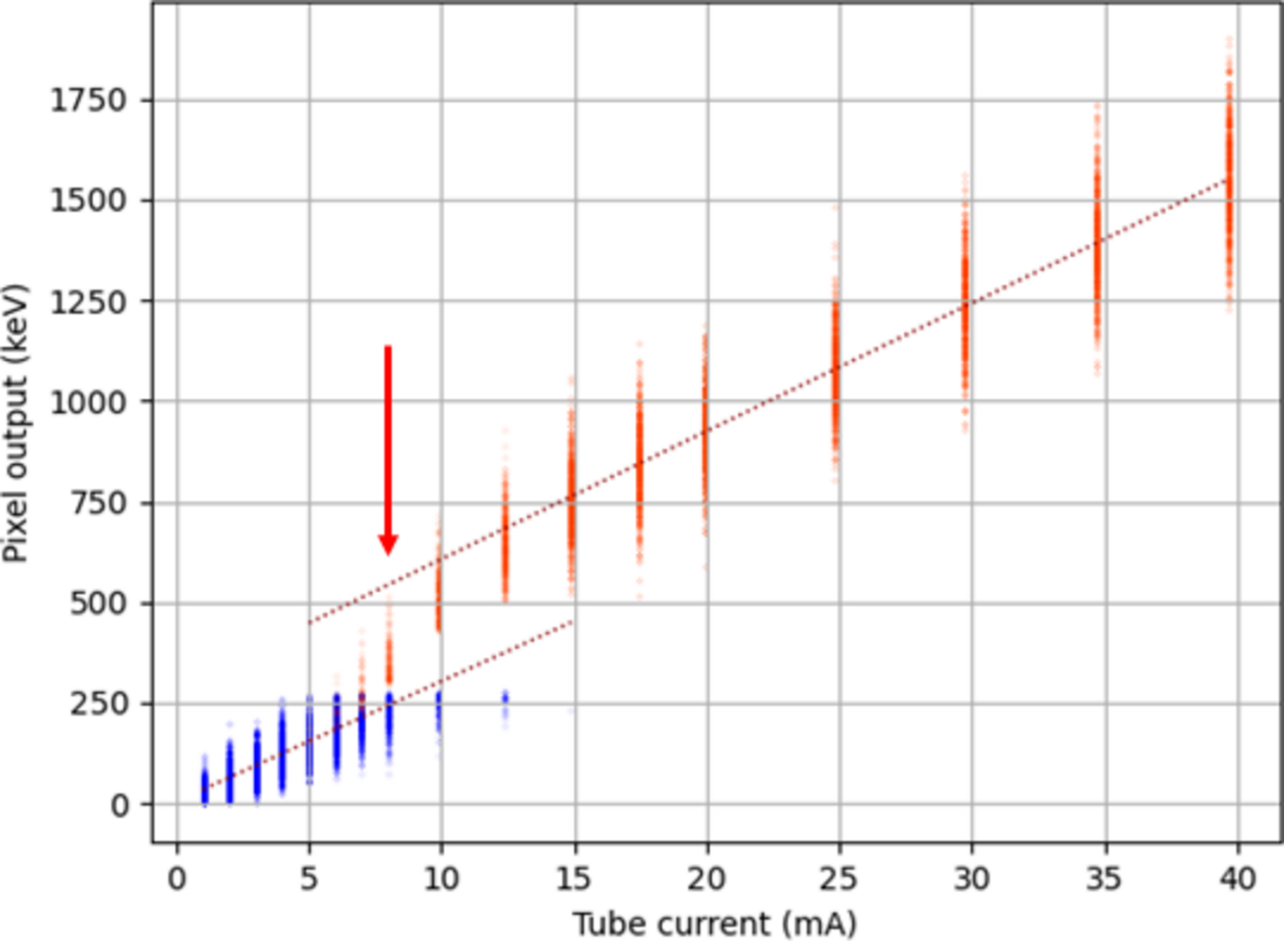
Output response after pedestal and gain corrections, for a single pixel with flood illumination (8 ASICs), showing a discontinuity around the point of switching from G0 to G1. In this case, below 6 mA the pixel is always in G0 (blue), above 15 mA the pixel is always in G1 (orange). Points within the gain change region were excluded from the fits. The point at which the discontinuity between the two gains was evaluated (8 mA tube current) is marked by an arrow.

A small group of pixels was selected near the corner of the detector module under test. Data were analysed for a subset of pixels in this area. Fig. 11 ▸ shows the behaviour of a typical pixel. The gain change between G0 and G1 took place in a region centred on 7–8 mA tube current. The vertical spread on the points at each selected beam current is due to Poisson variation in the number of photons per frame incident on the detector and the energy spread of the photons. The detector starts in G0 and, as the X-ray tube current increases, frame-to-frame fluctuations in the incident photon flux start to trigger the automatic gain change into G1. Eventually, the pixel is switched into G1 for all frames. For a perfect detector, the two gain regions should make up a continuous and linear relationship between the X-ray tube current and the pixel output. However, it was found that, in the region in which the gain was switching, there is a clear nonlinearity in the pixel response.

Two factors were investigated as potentially affecting the linearity of the calibration of the pixels. The first was the total area of the illumination. The illuminated area of a detector will affect its power consumption and hence the voltage drops on the metal layers in the ASIC may change. The temperature of the ASIC may also change. The overall result may be pedestal shifts and/or calibration changes. The second factor was the comparison between continuous mode pedestal recording and interspersed mode pedestal recording. The measured pixels were near 100 rows in, from the ASIC wire bonds.

#### 3.2.3. Linearity of single pixels as a function of illuminated area and pedestal mode

To study the effect of the area of illumination on the linearity of the detector, a series of lead masks was used to vary the total illuminated area, from a 3.5 mm-diameter circular aperture, through varying numbers of ASICs, up to a single module (8 ASICs). For each mask, a series of images was recorded for X-ray tube currents between 1 mA and 40 mA. The integration time used was 1 ms, with a frame rate of 1000 fps. The coolant temperature was set to typically −10°C, resulting in a measured temperature near 12°C at the module ROB. Table 1 ▸ summarizes the various illumination conditions used.

**Figure 12:**
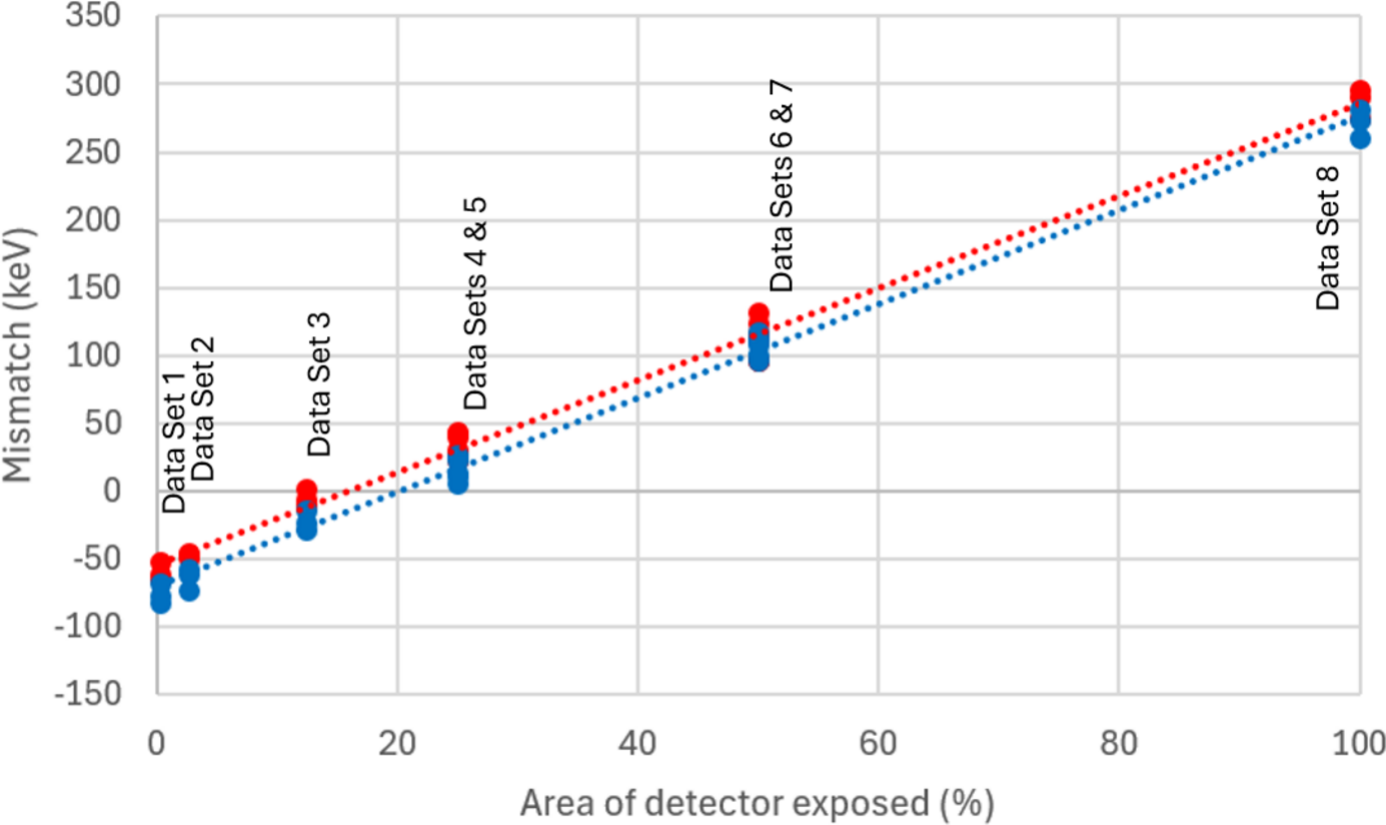
Size of the discontinuity at the switching point, versus area of illumination, for 3 individual pixels. Two data sets are shown: the first (red plot symbols) used continuous mode forced gain pedestals, the second (blue plot symbols) used interspersed mode. The data set index refers to different areas of illumination, as given in Table 1 ▸.

The nonlinearity in the response was quantified, by making a plot for each pixel, equivalent to Fig. 12 ▸. This plot was then divided into two regions: the first containing data for the pixel in G0 and the second for the pixel in G1. A separate linear fit was made to each region, excluding the area in which the pixel was sometimes in G0 and sometimes in G1. The expected pixel value near the centre of the switching region was calculated from each fit. The difference between the two values was taken to be a measure of the discontinuity in the pixel response. This value was than plotted as a function of the illumination area in Fig. 12 ▸.

From Fig. 12 ▸, it is apparent that the nonlinearity in the response of the detector scales with the area of illumination, in a way that is close to linear. There is only a small difference between the two methods of pedestal recording. The linearity may be explicable in terms of increasing the current draw of the ASICs, or parts of them, with corresponding drops in supply and reference voltages over the ASIC metal layers. There may also be temperature-related changes in the pixel behaviour. For the updated ASIC design, a similar measurement has been carried out with a single chip and will be repeated for an entire module in the future. The design changes are expected to reduce the nonlinearity seen here.

A correction algorithm is described by Biednov *et al.* (2023 ▸) for the application of Jungfrau detectors to hard X-ray emission spectroscopy. In this work, a von Hamos type spectrometer (Alonso-Mori *et al.*, 2012 ▸) was used with JF-1M area detectors. The target was a liquid jet and the X-ray illumination was provided by an XFEL, both factors that introduce significant frame-to-frame variation in intensity of the images recorded by the detectors. This allowed building up a calibration analogous to Fig. 11 ▸, for each pixel. Pixels which were illuminated at such a level that they remained in G0 throughout the data collection were used to provide a reference for the pixels which were switching.

The above method may have limitations, in that some pixels may not accumulate enough photon statistics to create a calibration. Also, it does not account explicitly for variations of the illuminated area, the importance of which is shown in Fig. 12 ▸. Nevertheless, the approach was demonstrated to work very well for the given application.

The size of the switching nonlinearity might be expected to change systematically with pixel position in the array and the total area of illumination (*e.g.* due to voltage drops or thermal effects). Future work with a laboratory-based X-ray source might be used to quantify this behaviour further, with the aim of developing a pixel-by-pixel correction which may be able to address the limitations above. Correction data might then be supplied with a new detector, in addition to the calibration which is supplied at present.

#### 3.2.4. Significance of the nonlinearity in the detector response

The significance of the nonlinearity was estimated by comparing it with the nominal switching point between the two gains; this is designed to take place near 30 photons of 10 keV energy, *i.e.* an integrated charge of 300 keV. The Poisson variation of 30 photons is near 5 photons, so the nonlinearity could start to affect the quality of the image once it exceeds 50 keV. This suggests that up to 25% of the module area could be illuminated before the nonlinearity becomes important.

For applications such as MX, the illumination is a diffraction pattern with sparse peaks. This type of application would be least affected by the nonlinearity in the detector response. For applications with a more diffuse image, the detector could deliberately be switched into intermediate gain mode. This would remove the switching discontinuity, at the expense of a poorer sensitivity to single photons.

## 4. Pedestal measurements using old and new methods

### 4.1. Long-term stability of forced gain pedestal measurements

The use of interspersed forced gain pedestal measurements was found to have little effect on the nonlinearity discussed in the previous section. However, it was considered that the use of interspersed forced gain dark frames to obtain mean pedestal values might also improve the long-term stability of the G1 and G2 pedestal measurements. Many dark runs were accumulated, in between protein crystallography measurements on beamline I24, and they were used to evaluate this.

The results presented here refer to 0.5 ms integration time throughout. Continuous dark frames were recorded at 100 fps and interspersed dark frames at 2000 fps. The times at which the dark runs were recorded were spaced irregularly throughout the day and the time elapsed between the first and last runs was between 22 and 24 h, in the figures shown.

The data were masked to remove the contribution of the larger sensor pixels, which are used to cover the gaps between the ASICs. Results are presented which average over the pixels of a single ASIC, rather than the complete module. Successive runs were concatenated. The behaviour of all the ASICs was very similar and so only a single example is shown.

**Figure 13:**
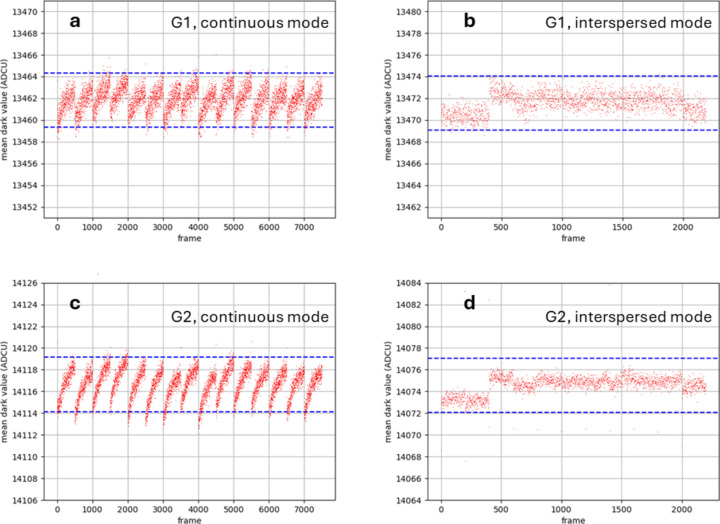
(*a*) Mean pedestal (ADCU) versus frame count for G1 dark runs, continuous mode (100 fps). (*b*) Mean pedestal (ADCU) versus frame count for G1 dark runs, interspersed mode (2000 fps). (*c*) Mean pedestal (ADCU) versus frame count for G2 dark runs, continuous mode (100 fps). (*d*) Mean pedestal (ADCU) versus frame count for G2 dark runs, interspersed mode (2000 fps). G1 gain is −1.36 ADCU per keV, G2 gain is −0.107 ADCU per keV.

The G1 pedestals recorded continuously at 100 fps for 0.5 ms integration time showed a clear sawtooth effect, associated with stitching the data files together [Fig. 13 ▸(*a*)]. This was most likely due to heating of the ASICs during the forced gain measurements, which is a known issue. The sawtooth for 0.5 ms integration time was near 6 ADCU peak-to-peak (the blue dotted lines are 5 ADCU apart, throughout Fig. 13 ▸), which corresponds to 4.4 keV, or 0.35 photon (at 12.4 keV). For comparison, the switching point between G0 and G1 occurs near 25 photons, so the sawtooth effect was small compared with the Poisson noise inherent in the illumination. There were some slow drifts in the G1 pedestals, although these were small.

The G2 pedestals recorded continuously [Fig. 13 ▸(*c*)] showed a more pronounced thermal sawtooth than was the case for the G1 pedestals. This was to be expected, as the G2 forced gain mode uses more power than the G1 forced gain mode, leading to increased temperature fluctuations for the ASICs. The peak-to-peak variation was again near 6 ADCU, which is equivalent to 60 keV. This is near 5 photons (at 12.4 keV). The switching point between G1 and G2 occurs near 900 photons, which has a Poisson noise of 30 photons; again the variation was small compared with the Poisson variation.

The G1 pedestals recorded using the interspersed method [Fig. 13 ▸(*b*)] showed an improvement as compared with the continuous method. There was no evidence of a thermal sawtooth and the overall envelope of the values was somewhat reduced. The average dark values were stable over 22 h.

The G2 pedestals recorded using the interspersed method [Fig. 13 ▸(*d*)] were also improved, compared with the continuous method. There was some evidence of jumps between data files, due to temperature variations from run to run. The overall envelope of the measurements lay close to a band of ±2 ADCU from the mean, *i.e.* ±20 keV. This is a clear improvement on the old method of recording forced gain pedestals.

The first forced gain frame in interspersed mode was always an outlier. In Fig. 13 ▸(*d*) these points can be seen near 14071 ADCU. In calculating pedestal values to be subtracted from data runs, these anomalous frames should be omitted. A similar problem, encountered previously with continuous mode pedestals, was eventually cured by a firmware update from PSI.

The absolute pedestal value changed by +10 ADCU (+7.35 keV, 0.6 photons), moving from continuous to interspersed gain in G1 and by −44 ADCU (−440 keV, −35 photons), moving from continuous to interspersed gain in G2. This implies that using G2 forced gain values from continuous recording instead of interspersed recording would introduce an error that is of similar magnitude to the Poisson variation at the point of gain change between G1 and G2.

Overall, it is reasonably clear that the interspersed method is an improvement on the continuous method. However, the error that is introduced by continuous recording would be unlikely to cause significant degradation to standard MX measurements.

## 5. Beamline measurements with the Jungfrau-1M

### 5.1. Single-photon detection on the beamline

**Figure 14:**
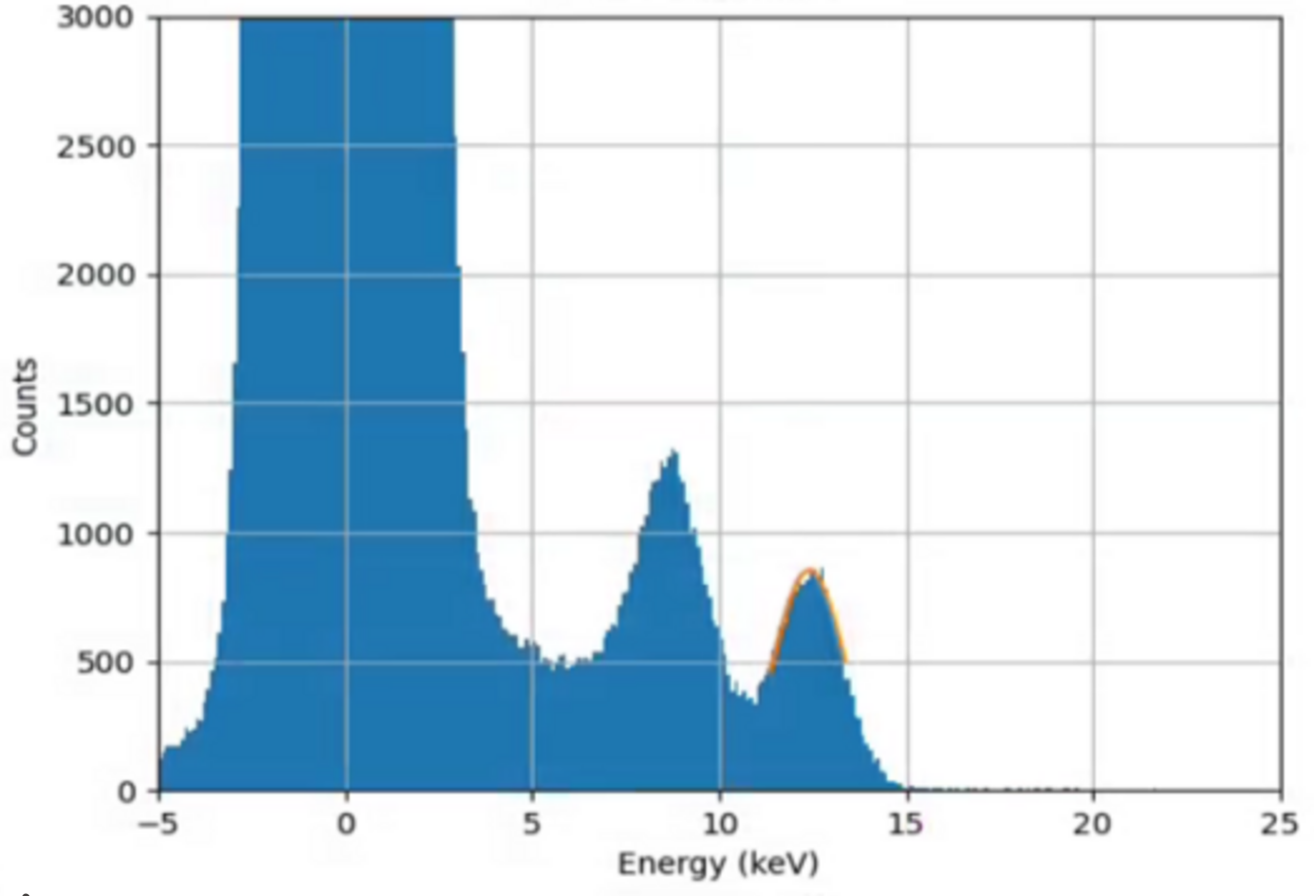
Spectrum from scattering a 12.4 keV beam using a glass sample. The 8.6 keV peak is due to the presence of zinc in the glass sample used.

The environment of a beamline is liable to be subject to more electrical noise than the detector test laboratory. It was decided to examine the sensitivity of the Jungfrau-1M to single photons on the beamline, to see if the good performance seen in the laboratory was preserved. A beam energy of 12.4 keV was used, with glass as a scattering material. An illuminated run was chosen in which the beam was attenuated to give a low flux at the detector, with integration time 1 ms and frame rate 1000 fps. The images from the Jungfrau-1M were corrected by pedestal subtraction and the application of the relevant gains, and noisy pixels were masked. A histogram was made of the resultant pixel values, and is shown in Fig. 14 ▸. Two peaks were seen in the spectrum and these were fitted with Gaussians. The peak energies were reconstructed as 8.62 keV and 12.38 keV. The former is consistent with being from the Zn *K*α fluorescence line, whilst the latter is consistent with the beam energy. ZnO is known to be a component of commercial glass.

The occurrence of two peaks prompted a check to ensure that the second peak was not due a thermal drift affecting the first. A data and a pedestal run were performed under nominally identical conditions, so that any thermal differences should be minimized, if the same frame numbers were selected for pedestal subtraction from the two files. To investigate the size of any thermal effects, 40 frames of data were taken from early in the illuminated run and compared with 40 frames of pedestal data from late in the dark run. This was then repeated vice versa, although different groups of frames were used in each case. The peak positions were 8.62 ± 0.02 and 12.38 ± 0.02 keV for matched data and pedestals. This can be compared with a known beam energy of 12.40 keV. Mismatching the illuminated and dark frames in time induced changes in peak position of up to ±0.4 keV, but no change in the shape of the spectrum. These results imply that single-photon detection in G0 mode is effective on the beamline as well as in the laboratory.

### 5.2. Distribution of single pixel values in a diffraction pattern

Diffraction patterns were recorded on Diamond beamline I24, from standard samples of insulin, using a series of beam attenuators to map out the dynamic range and linearity of the detector. The beam energy was 12.4 keV. Histograms were plotted of single pixel values, for diffraction images after dark subtraction and gain correction.

**Figure 15:**
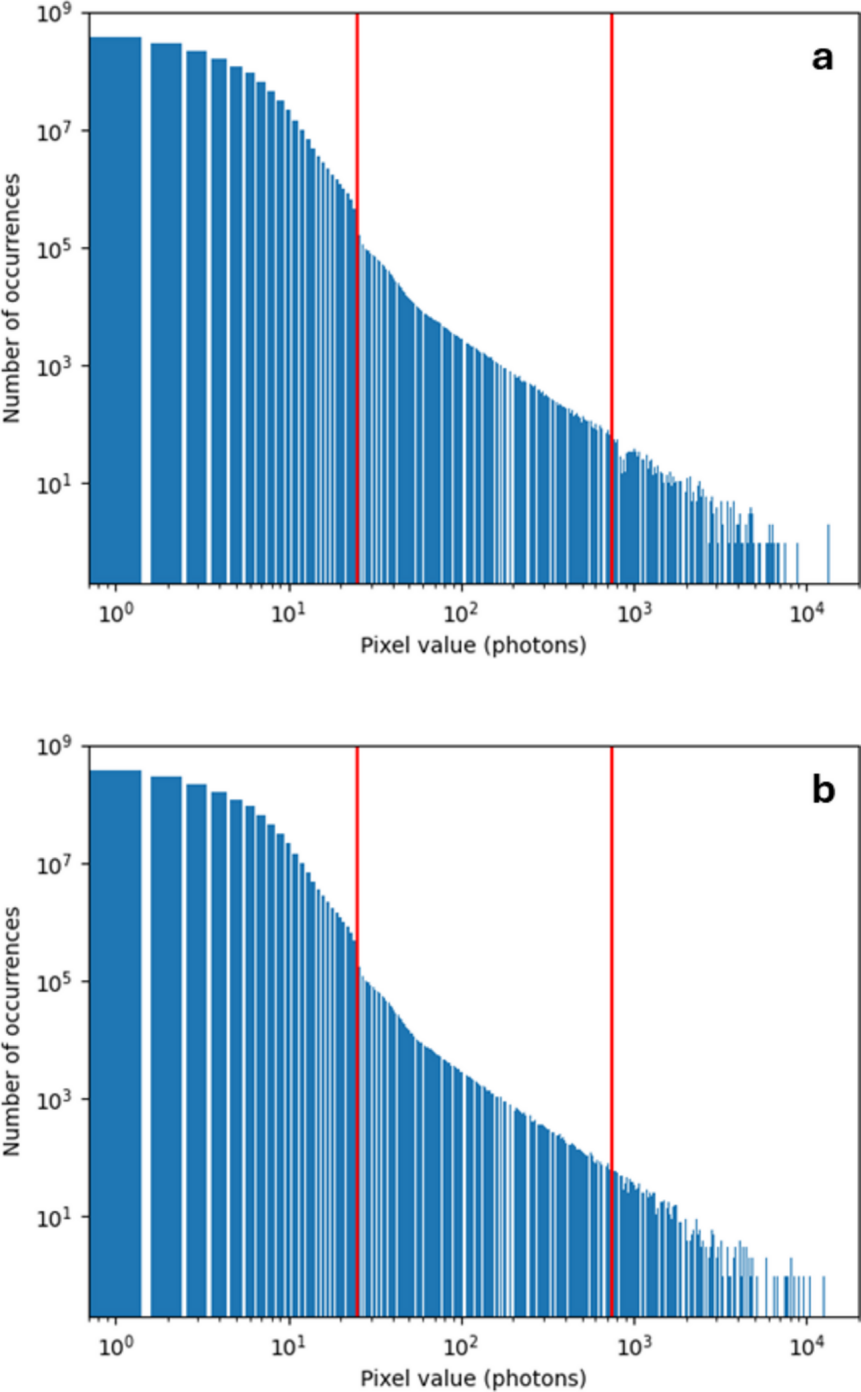
Histograms of all pixel values from an insulin diffraction pattern. (*a*) Using continuous mode pedestals. (*b*) Using interspersed mode pedestals. Integration time 0.5 ms, frame rate 2000 fps. The red lines mark the approximate gain switching points at 25 and 750 photons (12.4 keV).

Fig. 15 ▸ shows results with the beam attenuator set to give 100% transmission. The integration time of the detector was 0.5 ms, at a frame rate of 2000 fps. Matching dark runs for pedestal calculation were recorded at regular intervals on the beamline, with both continuous and interspersed modes.

The same G0 pedestal data were used throughout, and the analysis was repeated for forced gain pedestals recorded in both continuous mode [Fig. 15 ▸(*a*)] and interspersed mode [Fig. 15 ▸(*b*)]. The pedestal measurements used in continuous mode were made at 100 fps. The pedestal measurements used in interspersed mode were selected to match the beamline experiment, at 2000 fps. The integration time was 0.5 ms for all the data shown here. Both histograms show a discontinuity in the distribution, near 25 photons, which is the G0-to-G1 switching point for 12.4 keV. It was found that any differences between the two histograms were very small. This suggests that there was no significant improvement due to using interspersed mode pedestals in place of continuous mode pedestals. Variation of the attenuator values was found to move the end-points of the distributions in Fig. 15 ▸; however, no effect was found on the position of the discontinuity, which remained at 25 keV regardless of attenuator setting. This is further evidence that the discontinuity is an artefact coming from the detector and from the gain switching in particular.

### 5.3. Performance as a detector for MX

#### 5.3.1. Quality of MX diffraction data

The Jungfrau-1M was used to capture diffraction data from samples on DLS beamline I24. Diffraction data were taken from human insulin crystals prepared following standard protocols, with the samples kept at 100 K during data collection to minimize radiation damage. The data (https://zenodo.org/records/15017658) were recorded following the NXmx convention (https://www.nexusformat.org/). The mode of data capture resulted in data files structured differently to the HDF5 structures used by, *e.g.*, DECTRIS. However, the HDF5 ‘virtual data set’ feature was used to restructure the data following the standards. The data were taken with the detector 66 mm from the sample, at a wavelength of 0.8856 Å, 25.8% transmission with 0.1° per frame, 360° total with a total exposure of 1.8 s.

The data were processed following standard protocols, using the software *DIALS* (Winter *et al.*, 2018 ▸), as detailed in https://github.com/graeme-winter/dials_tutorials/blob/main/ccp4-aps-2024/WORKFLOW.md.

The key processing steps were:

(i) Location of the diffraction maxima (‘spots’).

(ii) Derivation of the crystal unit cell and lattice from the positions, assignment of the Miller indices for each diffraction spot.

(iii) Refinement of these models.

(iv) Application of these models to ‘integrate’ the data (*i.e.* compute the number of photons contributing to unique spots, subtracting an estimate of the local background).

(v) Correction of the data to account for experimental effects, and merging of equivalent data giving statistics on the reproducibility of the measurements.

Throughout this process, diagnostics were available from the software, to indicate the level of agreement between the observed data and the mathematical models used to analyse them (*e.g.* the typical distance between predicted and measured spot positions). These give guidance on the sensitivity of the detector, the linearity and the accuracy of assembly.

In the initial spot finding stage, some 170000 or so spots were found across the whole data set, with a few hundred strong pixels on every image. The analysis of these positions assigned indices to over 98% of the spots, indicating that there were essentially no artefacts in the data (*i.e.* false spots, resulting from poor pedestal modelling), and refinement of this model gave a measure of alignment between the observed and calculated spot locations of 0.12, 0.10, 0.14 pixels for the fast and slow directions on the detector and the frame number, respectively. For reference, these are unusually good RMS deviations – between 0.2 and 0.5 pixels would be typical.

After integration and scaling the errors are adjusted to ensure consistency between the uncertainty of individual intensities and the spread of symmetry-related reflections. For these data, the scale factor applied was 0.95 with a term proportional to the intensity of 0.03 giving an (*I*/σ)^asymptotic^ (Diederichs, 2010 ▸) of around 35. This is consistent with data recorded on I24 with a photon-counting detector.

In essence, this indicates a ∼3% systematic error, but the overall scale applied to the uncertainties is about 1, indicating that the data are effectively Poissonian, This supports the expectation of individual photon sensitivity. Overall, the scaled data agree very well with one another, as highlighted by the typical MX experiment ‘Table 1’ (shown here as Table 2 ▸).

**Figure 16:**
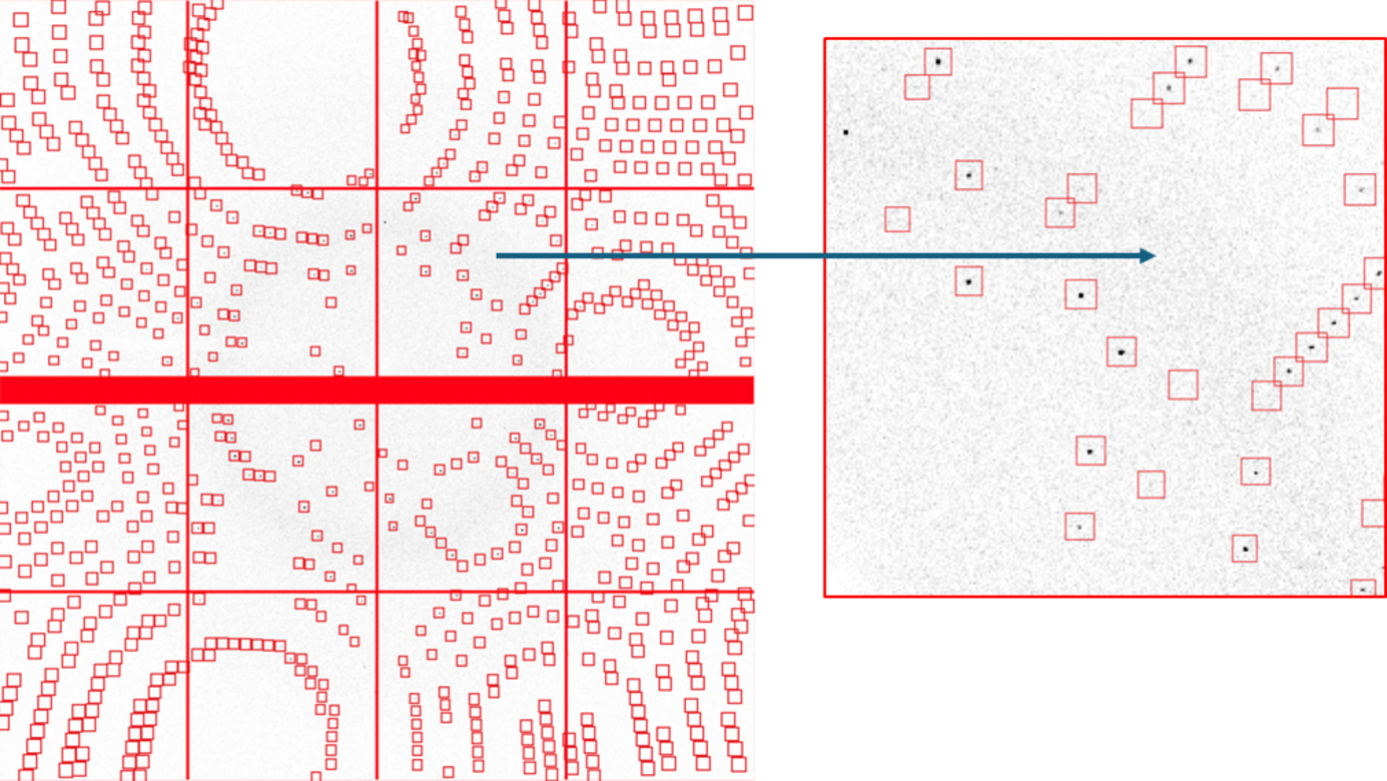
Image obtained with the Jungfrau-1M detector, showing the indexed diffraction spots. The zoomed-in area corresponds to a single ASIC.

The raw data are presented in Fig. 16 ▸, which shows the overall image with the masked pixels (the inter-module gap and ‘big’ pixels) with the processing boxes. These should be around three times the size of the spots in each direction to allow area to estimate the local background. The zoom shows a single ASIC, with the intention being to show that (i) the spots are very sharp and (ii) the background between spots is very uniform (between 0 and 2 photons, typically), indicating once more that the cooling of the detector gives rise to a very low integration noise. It is noteworthy that negative pixels are very rare, indicating that the pedestal estimates are reliable.

#### 5.3.2. Linearity

Rotation data were captured for the same insulin crystal over several runs, increasing the attenuator transmission each time. The resulting data were again processed using *DIALS* (Winter *et al.*, 2018 ▸). *DIALS* was used to find, index and integrate reflections. Data were acquired, varying attenuation in the beam from run to run. Indexed reflections were compared between runs, giving an indication of the linearity and noise of the reflections after processing.

**Figure 17:**
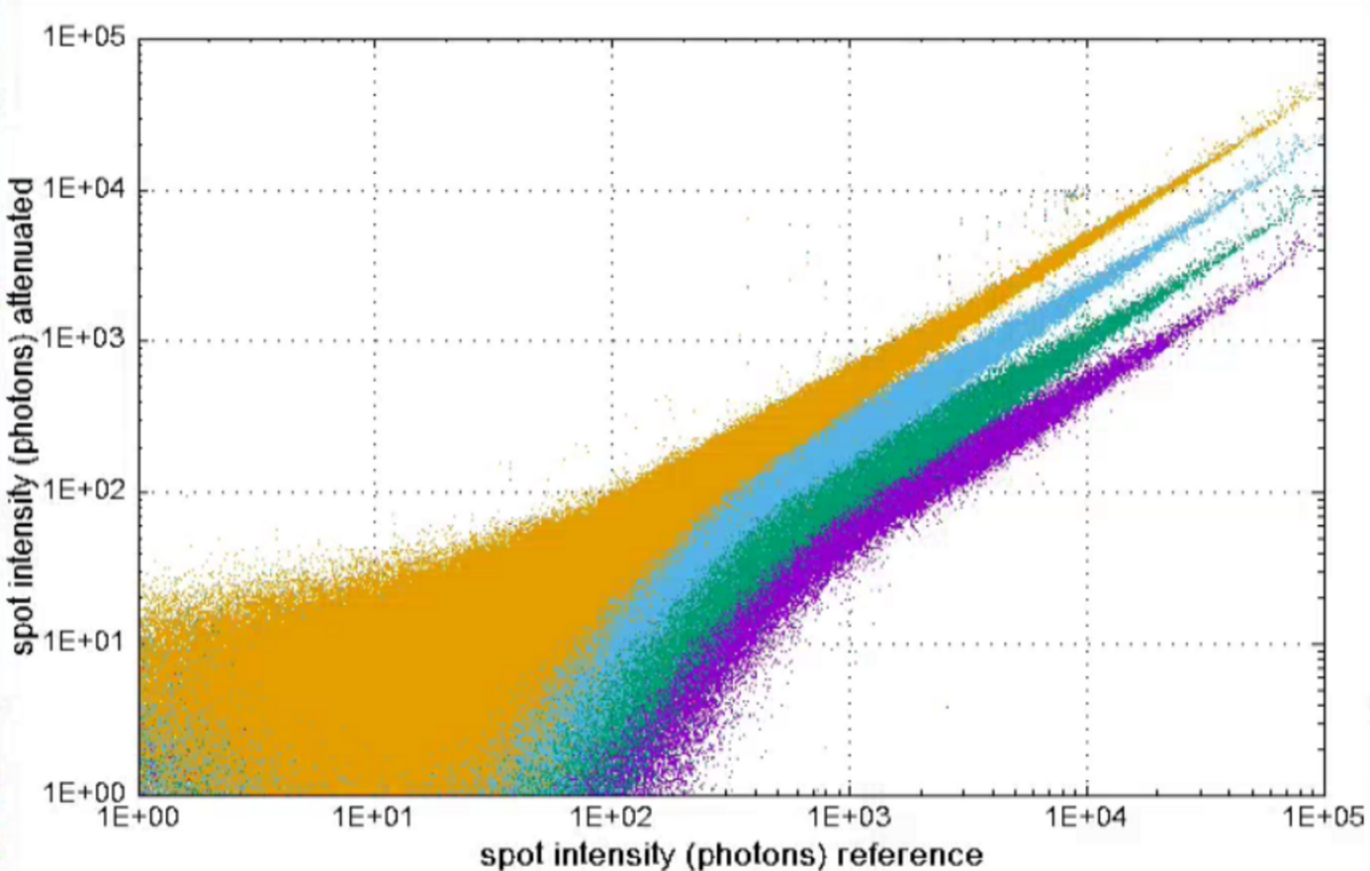
Linearity from MX measurements on the beamline. Transmissions were 50% (amber), 25% (blue), 12.5% (green) and 6.25% (purple).

Fig. 17 ▸ shows data from a single insulin crystal. Rotation scans, each with a different level of attenuation, have been plotted against a reference scan with 100% transmission. The reflections were integrated using the profile fitting algorithm of *DIALS*. The integration typically occurs over several pixels in each spot, so that the nonlinearities seen in single pixel data (*i.e.* in the histograms of Fig. 15 ▸) are not visible in this processed MX data. The data sets scaled according to the attenuation values.

## 6. Discussion

A Jungfrau-1M detector was evaluated at Diamond Light Source, both in an offline laboratory and at beamline I24. Jungfrau is an integrating detector and represents a move away from the ubiquitous photon-counting approach. This move has been made necessary by the increasing flux at XFELs and synchrotron light sources. Diamond itself is scheduled to undergo a major upgrade, with work beginning in 2025 and continuing over several years. This will result in an increase in the photon energy and flux at many beamlines, including I24. At I24, the maximum incident flux at the detector can already be made high enough to make the use of an integrating detector advantageous.

The operation of Jungfrau entailed some differences, compared with the existing (counting) beamline detectors. The sensor and front-end electronics need to be kept cool, to suppress leakage currents which are otherwise integrated and may affect the signal measurement. We found that a coolant temperature of −10°C, for a ROB temperature near +12°C, was adequate. (The front-end electronics are expected to be close in temperature to the coolant flow, by design.) The stability requirement of the front-end temperature is also more stringent for an integrating detector than for a counting detector, but is within the capability of standard off-the-shelf laboratory chillers. Pedestals need to be subtracted from data from integrating detectors and we found that the pedestal behaviour of the Jungfrau-1M was both stable and reproducible over experimental timescales, given adequate temperature control.

The signal-to-noise ratio of an integrating detector is maximized by keeping the integration time as short as possible, which results in high frame rates and therefore places unusually high demands on network bandwidth. During this work, we operated the Jungfrau-1M at up to 2000 fps, which gives a data rate of 32 Gb s^−1^. This scales with detector pixel count and is a driving factor behind the need for data processing and reduction, in parallel to data acquisition. The necessity to subtract dark images and to apply gain corrections also makes it advantageous to be able to process data in parallel, enabling (*e.g.*) a live display for the user.

The Jungfrau-1M was found to be capable of detecting single photons, with good signal-to-noise, at 12.4 keV on I24 and using 8 keV fluorescence photons in the laboratory. The good signal-to-noise implies that the detector is capable of imaging single photons at lower energies than this. However, for our application, higher photon energies are envisaged for the future of Microfocus MX at Diamond-II (Storm *et al.*, 2021 ▸). The maximum flux measurable with the detector was near 1 × 10^4^ photons per pixel per frame, equivalent to 4 × 10^9^ photons s^−1^ mm^−2^ at 2000 fps. In between these extremes, it was found that the response of the detector had good linearity, except for a region around the first gain switching point. This was visible as a discontinuity in histograms of beamline single pixel data and in calibration curves obtained for single pixels using the laboratory X-ray set. It was unchanged by a change in pedestal recording technique, which was intended to reduce heating in the ASIC during the recording of forced gain pedestals. Nonetheless, integration of diffraction spots led to an averaging effect, and the single pixel discontinuity gave no visible degradation to the linearity, as measured using integrated MX spots. It is expected that this discontinuity will be reduced in the most recent version of the Jungfrau ASIC (V1.2). The linear variation of the discontinuity with area of illumination may also offer the possibility of developing a correction algorithm, similar to the one given by Biednov *et al.* (2023 ▸), but ideally based on an offline laboratory X-ray calibration.

Analysis of MX data from rotation scans with insulin showed no artefacts (false spots) in the data. Alignment between calculated and observed spot positions was very good and the data were effectively Poissonian, in line with the measured single-photon sensitivity.

## Figures and Tables

**Table 1:** Key to the illumination conditions in Fig. 12 ▸

Data set	Conditions	Approximate area exposed
1	3.5 mm-diameter circular mask	0.3%
2	10 mm-diameter circular mask	3%
3	1 ASIC	12.5%
4	2 ASICs horizontally	25%
5	2 ASICs vertically	25%
6	1 × 4 ASICs	50%
7	2 × 2 ASICs	50%
8	Flood illumination	100%

**Table 2:** Scaling and merging statistics for insulin, from diffraction data recorded using the Jungfrau-1M

	Suggested	Low	High	Overall
High-resolution limit (Å)	1.31	3.57	1.31	1.28
Low-resolution limit (Å)	38.91	38.93	1.34	38.91
Completeness (%)	96.1	100.0	70.2	92.0
Multiplicity	28.7	36.1	4.7	27.9
*I*/σ	21.1	113.8	0.3	20.5
*R*_merge_(*I*)	0.084	0.035	1.857	0.084
*R*_merge_(*I*±)	0.083	0.035	1.653	0.083
*R*_meas_(*I*)	0.085	0.036	2.081	0.085
*R*_meas_(*I*±)	0.085	0.035	2.052	0.085
*R*_pim_(*I*)	0.014	0.006	0.903	0.014
*R*_pim_(*I*±)	0.020	0.008	1.179	0.020
CC half	1.000	1.000	0.280	1.000
Anomalous completeness (%)	94.9	99.5	62.9	89.8
Anomalous multiplicity	15.0	19.9	2.5	14.6
Anomalous correlation	0.094	0.051	−0.004	0.108
Anomalous slope	0.578			
d*F*/*F*	0.051			
d*I*/*s*(d*I*)	0.539			
Total observations	517887	36312	2998	519530
Total unique	18053	1006	644	18646

## Data Availability

The data recorded on Diamond beamlines can be supplied by request from the authors. An example MX data set from this work is available at https://zenodo.org/records/15017658.
